# Empirical Scenarios of Fake Data Analysis: The Sample Generation by Replacement (SGR) Approach

**DOI:** 10.3389/fpsyg.2017.00482

**Published:** 2017-04-19

**Authors:** Massimiliano Pastore, Massimo Nucci, Andrea Bobbio, Luigi Lombardi

**Affiliations:** ^1^Department of Developmental and Social Psychology, University of PadovaPadova, Italy; ^2^Department of General Psychology, University of PadovaPadova, Italy; ^3^Department of Philosophy, Sociology, Education, and Applied Psychology, University of PadovaPadova, Italy; ^4^Department of Psychology and Cognitive Sciences, University of TrentoRovereto, Italy

**Keywords:** sample generation by replacement, fake data, self-report measures, monte carlo, scenario-based methodology

## Abstract

Many self-report measures of attitudes, beliefs, personality, and pathology include items whose responses can be easily manipulated or distorted, as an example in order to give a positive impression to others, to obtain financial compensation, to avoid being charged with a crime, to get a job, or else. This fact confronts both researchers and practitioners with the crucial problem of biases yielded by the usage of standard statistical models. The current paper presents three empirical applications to the issue of faking of a recent probabilistic perturbation procedure called Sample Generation by Replacement (SGR; Lombardi and Pastore, 2012). With the intent to study the behavior of some statistics under fake perturbation and data reconstruction processes, *ad-hoc* faking scenarios were implemented and tested. Overall, results proved that SGR could be successfully applied both in the case of research designs traditionally proposed in order to deal with faking (e.g., use of fake-detecting scales, experimentally induced faking, or contrasting applicants vs. incumbents), and in the case of ecological research settings, where no information as regards faking could be collected by the researcher or the practitioner. Implications and limitations are presented and discussed.

## 1. Introduction

In psychology and social sciences self-report items are commonly used to measure personal attitudes and beliefs and it is well-known that, depending on the context, they can be easily manipulated by respondents as they may know, or can easily imagine, what are the correct, or socially desirable, or more convenient answers, even if that answers do not match their own true intentions (Ziegler et al., 2012). Several reasons may push people to fake, e.g., in order to appear better than one actually is (Furnham, 1986; Paulhus, 1991; Zickar and Robie, 1999; McFarland and Ryan, 2000), to dissimulate vocational interests, to pass an exam, to be recruited by a headhunter or by a prestigiuos college, to simulate grossly exaggerated physical or psychological symptoms as a way to obtain financial compensation, to avoid being charged with a crime, to obtain drugs, to have access to advantageous positions (e.g., being a good candidate for a transplant) or, possibly, simply because contrary to the purpose of research (e.g., Mittenberg et al., 2002; Hall and Hall, 2007). In these sensitive contexts the issue of data faking may have an influence on experimental results or, quite disturbingly, also in relation to test validation. Therefore, it plays an important role in areas like psychology (e.g., Hopwood et al., 2008), education (Burrus et al., 2011), work and organizational psychology (e.g., Van der Geest and Sarkodie, 1998), medicine (especially in the case of forensic medicine/psychology; e.g., Gray et al., 2003), and many others.

Detecting, understanding, and managing faking is difficult when researchers or practitioners rely only on traditional data analysis strategies. Field research has provided a variety of methods for studying faking which are, following Reeder and Ryan (2012), roughly distinguishable into two categories: proactive and reactive approaches. The proactive approach aims to reduce motivation to fake or to increase the difficulty of successfully cheating. Randomized response techniques (Warner, 1965; Tracy and Fox, 1981; Hussain et al., 2007), for example, represents a tangible way through which the subject can experience a situation in which his/her answer could not be related to his/her actual opinion or action. Additionally, making the goal of a test not immediately evident to the respondent is another possibility. Placing the items belonging to a specific dimension (e.g., a personality trait) distant from each other, or reversing their meaning or adding irrelevant items (for disguise purposes), and so on, are other examples of proactive techniques. Finally, using warnings that led the respondent to believe that the experimenter may have the ability to detect fakers and/or threatening fakers with their exclusion from the testing situation, are further examples of proactive techniques.

Reactive approaches include methods to counteract the effect of faking after the measure have been administrated and/or data have been collected. A wide collection of techniques, based on psychological models or statistical expectations, are used in order to identify individual cases or groups of respondents who are suspected to have distorted responses (and variance in scores) in a way that could be attributed to faking. After detection a number of correction strategies could be employed.

Administration of social desirability scales is one of the most common methods used in order to highlight people who are more inclined to lie (Leite and Cooper, 2010; Ferrando and Anguiano-Carrasco, 2011); the presence of some “bait-and-switch items” for liars (e.g., “I know all studies made by Dr. Sempronio,” who is, of course, an imaginary researcher) is a reactive technique. Still, several techniques have been proposed with the aim to detect inconsistencies (e.g., “I never remember my dreams” and, subsequently, “I dream of things which are not complicated”). Newer and more advanced data analyses techniques have been devised in order to study the “structure” of group data, or patterns of individual responses, which could be considered as less likely to be found, e.g., in terms of suspicious covariance, and possibly using and combining these suspects with other measures, e.g., time latency (Ziegler et al., 2012). Obviously all the techniques mentioned above have limitations and their critical discussion is not a goal of the present paper: for more details, please see Ziegler et al. (2012). In any case, reactive techniques are based on the need to identify faking behaviors, and, at least, they require one of the two following circumstances: (a) a scale—or at least some items—devised in order to catch fakers and/or, (b) a specific model of detection and data analysis able to grasp internal aberrancies. In the latter case, a reliable dataset to be compared with the suspicious one is required.

In conclusion, as far as we know, no methodology has been put forward with the explicit intent to deal with situations in which the researcher does not have available (or cannot use) a dedicated lie scale, or a sample of reliable data to be compared to the doubtful dataset is not handy, with few recent exceptions referring to the Item Response Theory (IRT) (Mneimneh et al., 2014; Falk and Cai, 2016).

In this paper, we present a reactive approach based on an adaptation of a recent probabilistic procedure named *Sample Generation by Replacement* (SGR; Lombardi and Pastore, 2012; Pastore and Lombardi, 2014). Usually, SGR can be used with discrete items and without any specific fake-detecting scale, and it can also be applied with many different types of statistical analysis (e.g., Lombardi and Pastore, 2012, 2014; Pastore and Lombardi, 2014). However, SGR is not specifically designed for detecting data faking, but rather to try to answer the following empirical question: If data included fake observations, to what extent would the empirical results to the research questions be different from what they actually are? In other words, which percentage of fake data observations within a data set would lead the results (e.g., parameter estimations, model fit evaluations) to be somehow different from what they actually are? Moreover, when some information (empirical or hypothetical) about the presence of fakers is available, the SGR approach could also be used as a way to implement correction strategies in order to reconstruct the hypothetically true unobserved data. However, for this latter purpose, a simple modification of the standard SGR procedure is necessary.

The paper, which has mainly a methodological purpose, is organized as follows: we start with a brief introduction of the main tokens of the SGR approach, as it was originally described by Lombardi and Pastore (2012) and then we illustrate a simple SGR modification, called R-SGR (Reversed SGR), to optimally cope with the data faking problems studied in this article. Subsequently we present three empirical applications of the R-SGR procedure to fake data analysis. In the first one, which was planned as a way to compare our proposal with alternative methodologies to deal with faking, we followed a data collection strategy that reflects the approach described by Ferrando and Anguiano-Carrasco (2011): that is, a group of fake-motivated respondents is contrasted with a non-faking motivated one, within a repeated measures design. The second application is similar to the first one, but the repeated measure design was not adopted. The third application aimed to reproduce an highly realistic or “ecological” situation: only one sample with the suspected presence of fakers was used, and no other information was available. This could be the case of a researcher administering a psychological scale only once to a group of students attending an introductory course at College, or that of a professional psychologist managing a selection process where psychological tests are administered without any control group. Finally, conclusions and some relevant comments about limitations, potential new applications, and extensions of the SGR approach are discussed.

## 2. From SGR to reversed SGR

Standard SGR is based on a two-stage sampling procedure which characterizes two distinct models. The first model defines the process that generates the data before any kind of data perturbation or manipulation (this is called the *data generation process*). The second model represents the process used in order to perturb the data (named the *data replacement process*). The logic behind the SGR approach can be summarized as follows: if we repeatedly sample data through the two-stage procedure, the resulting collection of simulated/artificial data sets can be evaluated on the basis of some relevant measures computed on these simulated data. In SGR the first process is modeled by means of standard Monte Carlo (MC) procedures for ordinal data, whereas the second process is implemented using *ad-hoc* probabilistic models (e.g., Lombardi and Pastore, 2012, 2014; Pastore and Lombardi, 2014)^1^. Up to now SGR has mainly been used to implement MC simulation studies and evaluate the sensitivity of statistical indices to possible fake data in multivariate models. However, when the reconstruction of hypothetical true information becomes the primary objective of a data analyst or researcher, then the basic SGR procedure needs to be modified to account for the natural reversing sequence of the reconstruction mechanism.

More precisely, with respect to the reversed fake-data problem we can describe the supposedly fake observed data as an *n* × *m* data matrix F, including *n* i.i.d. response patterns (i.e., the participants responses) described by *m* distinct elements (i.e., the self-report items). In SGR, we assume that entry f_*ij*_ of F (*i* = 1, …, *n*; *j* = 1, …, *m*) takes values on a small discrete ordinal set *V*_*q*_ = {1, …, *q*} (for the sake of simplicity, in this presentation we assume identical ordinal sets for all items). In particular, if we denote by f_*i*_ the (1 × *m*) array of F representing the observed response pattern of participant *i*, then the R-SGR procedure allows us to construct a new *n* × *m* ordinal data matrix D, called the hypothetical *true data matrix* of F, by transforming each element f_*ij*_ in F on the basis of a dedicated replacement probability distribution, that we call the *reversing distribution*. Moreover, let d_*i*_ be the corresponding (1 × *m*) array of the reconstructed data matrix D, then this response pattern is also required to be a multidimensional ordinal random variable, in other words, D and F share the same ordinal scale level. Finally, by repeatedly generating artificial data via the R-SGR mechanism, we can obtain a collection of reconstructed data sets and study the statistical properties of this collection representing the hypothetical characteristics of the (unobserved and unknown) fake-uncorrupted scenario.

### 2.1. The reversing distribution

To reconstruct the hypothetical true data, we adopted a reversing distribution to generate the new values which can be understood as being inversely related with the direction of a *faking process*. In our study, we limited our attention to the faking good scenario. The dual configuration based on faking-bad manipulations can be easily obtained by a straightforward modification of the reversing distribution and will not be discussed here (Lombardi and Pastore, 2014). More precisely, in SGR a fake-good manipulation always represents a context in which the responses are exclusively subject to positive feigning:

(1) $$\begin{array}{r} f_{ij}\geq d_{ij}\;i=1,\ldots ,n;j=1,\ldots ,m. \end{array}$$

Therefore, the reversing distribution can be described according to the following equation:

(2) $$\begin{array}{l} p\left(d_{ij}=h|f_{ij}=k,\theta_{R}\right)=\{\begin{array}{cc} 1, & h=k=1\\ DG(h;a,b,\theta_{R})\pi , & 1\leq h<k\leq q\\ 1-\pi , & 1<h=k\leq q\\ 0, & 1\leq k<h\leq q \end{array} \end{array}$$

**Figure 1:**
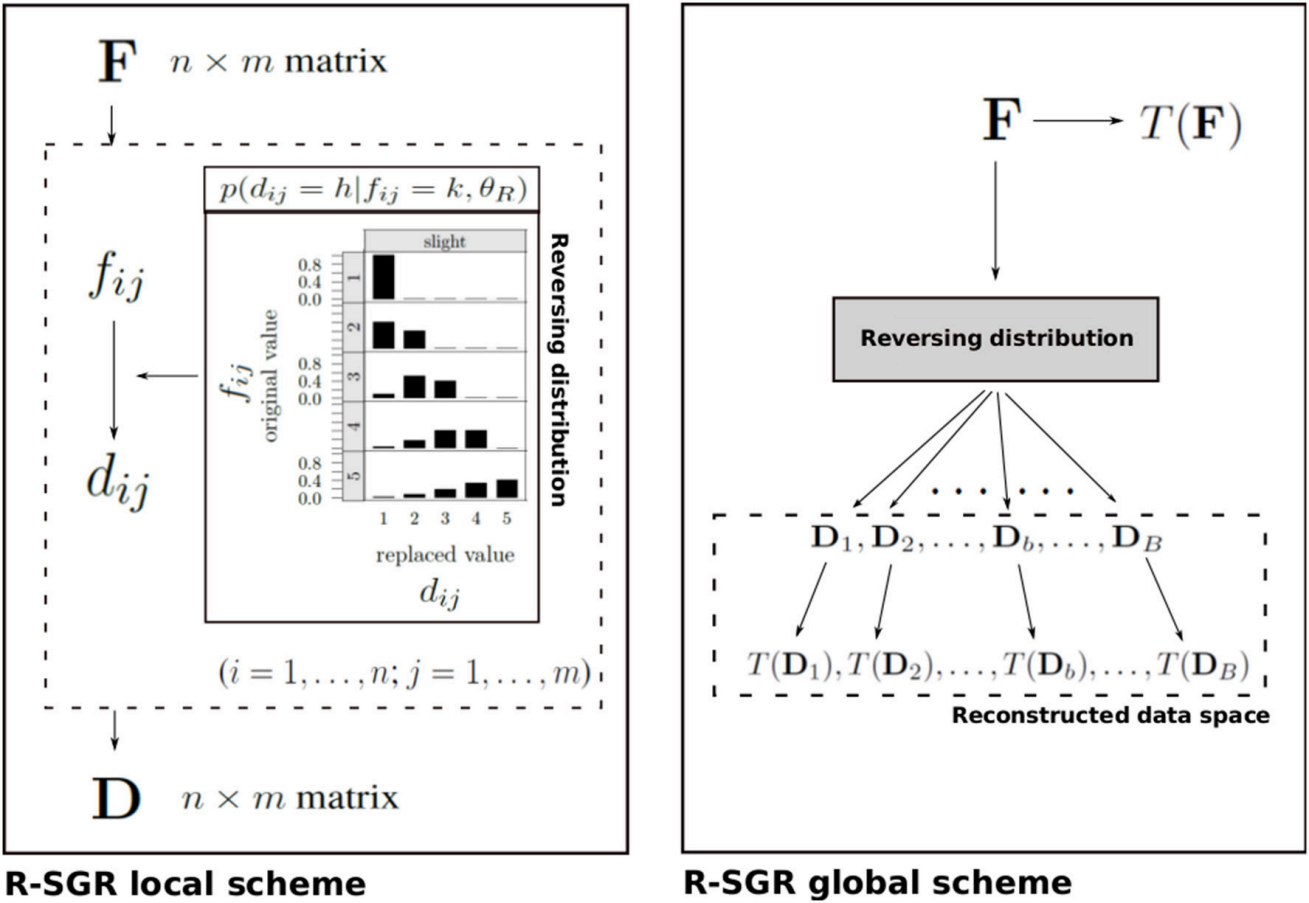
**Overall structure of the R-SGR procedure**. **(Left panel)** The R-SGR local scheme describes the generation of a reconstructed (*n* × *m*) true data matrix D on the basis of an observed (*n* × *m*) fake data matrix F with d_*ij*_ and *f*_*ij*_ being the (*i, j*)-entry of D and F, respectively. In this local scheme the reversing distribution (see Equation 2 in the text) is separately applied to each (*i, j*)-entry of F and mimics a slight faking good process. **(Right panel)** The R-SGR global scheme illustrates how the R-SGR procedure can be used to generate a simulated reconstructed data space as well as a corresponding simulated space of statistical outcomes *T*(*D*_*b*_) on the basis of a target statistic *T*. *T*(*F*) is the statistical outcome computed on the observed data F.

This equation represents the conditional probability of replacing an original observed value *k* in entry (*i, j*) of F with the new value *h* in the corresponding entry of the reconstructed data matrix D. In Equation (2), *DG* indicates the generalized beta distribution for discrete variables with range bounds *a* = 1 and *b* = *k*−1, respectively (for more details the reader can refer to Pastore and Lombardi, 2014), whereas θ_*R*_ = (γ, δ, π) is the parameter array which governs the behavior of the reversing model. Notice that in θ_*R*_, the parameters γ and δ constitute strictly positive shape parameters for the reversing distribution and modulate the way the shape of the distribution looks like. Finally, in Equation (2) the parameter π is understood as the hypothetical overall probability of a faking good process and plays the role of a rescaling weight for *DG*. Figure 1 depicts a general scheme of the R-SGR procedure.

**Figure 2:**
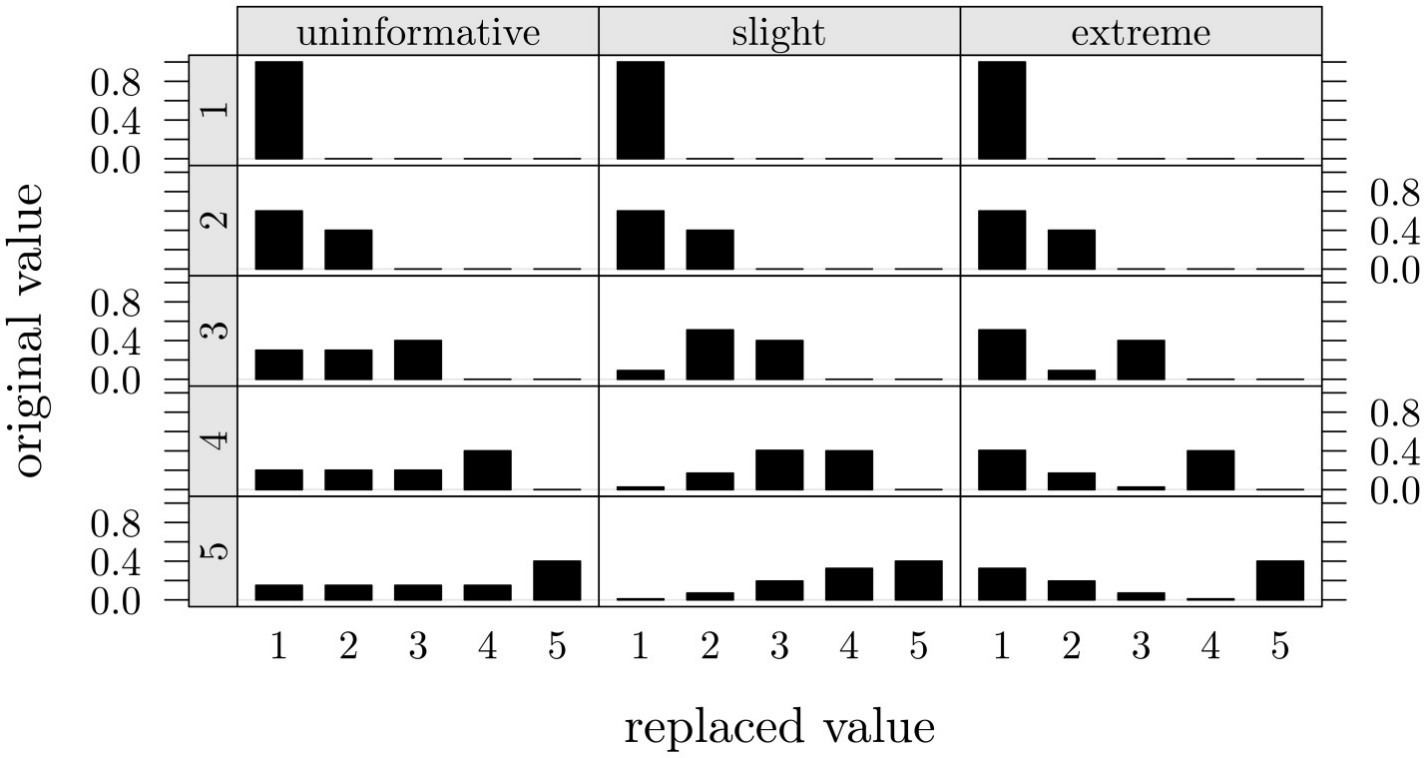
**Three models of conditional replacement distributions for a 5-point discrete r.v**. Each column in the graphical representation corresponds to a different conditional replacement distribution with overall probability of replacement π = 0.6 and one of the three different assignments for the shape parameters (uninformative: γ = δ = 1; slight: γ = 4, δ = 1.5; and extreme: γ = 1.5, δ = 4). Each row in the graphical representation corresponds to a different original 5-point discrete value *h*.

The reversing model represented in Equation (2) is very flexible as it can characterize both symmetric (Figure 2, first column) as well as asymmetric (Figure 2, second and third columns) reversing mechanisms in the SGR architecture. In particular, if γ = δ = 1 (Figure 2, first column), the model reduces to a reversing process based on a uniform random assumption such that in absence of further knowledge about the process of faking all entries in the observed data set F are assumed to be equally likely to be replaced with alternative values drawn from a discrete and uniformly distributed set of values. Figure 2 (first column) represents five examples of this type of uniformity condition in the reversing distribution.

By contrast, if we set the values of the shaping parameters as 1 ≤ γ < δ (resp. 1 ≤ δ < γ), then we can mimic asymmetric reversing distributions which identify mild positive shifts (resp. exaggerated positive shifts) in the value of the original observed response (Figure 2, second and third columns).

In particular, the condition γ < δ mimics an asymmetric reversing scenario in which the reconstructed value corresponds to a moderate shift in the value of the observed self-report response (*slight model*). In this model the chance to replace an original value *k* with another lower value *h* decreased as a function of the distance between *h* and *k*. This configuration can be applied whenever we believe that the observed data F has been corrupted by a slight faking process (Zickar and Robie, 1999; Zickar et al., 2004). Figure 2 (second column) shows five examples of reversing replacement distributions for the slight faking.

On the contrary, the condition γ > δ describes an asymmetric reversing scenario in which the reconstructed value corresponds to an extreme shift in the observed self-report value (*extreme model*). Unlike the slight model, in this alternative representation the chance to substitute an original value *k* with another lower value *h* increases as a function of the distance between *h* and *k*. The extreme model can be adopted if we believe that the observed data F has been corrupted by a sort of extreme faking process (Zickar and Robie, 1999; Zickar et al., 2004). Figure 2 (third column) depicts five different examples of reversing distributions for this kind of extreme faking representation.

Finally, a special case occurs when the parameter π is equal to 0. In this particular situation the reconstructed data matrix D simply boils down to the original observed data matrix F.

Generally speaking, the amount of faking may differ both among individuals as well as contexts or situations (Ziegler et al., 2012). In order to model the faking mechanism we adopted three *ad-hoc* levels of graded fake manipulations: uninformative/neutral faking, slight faking, and extreme faking. When specific information on participants or contexts are available, or also specific theories of personality from which assumptions on participants' response styles could be derived, other perturbation models can be easily implemented by modifying γ and δ parameters. In our case we decided to choose three simple but still relevant examples.

## 3. Empirical applications

As described before, the use of instruction sets represents the most common methodology adopted for investigating faking behavior. In particular, participants are usually asked to answer under a *respond honestly* or a *motivated distortion* condition (e.g., Ferrando, 2005; Ferrando and Anguiano-Carrasco, 2009; Leite and Cooper, 2010; Ferrando and Anguiano-Carrasco, 2011). In the first example (study one), we took inspiration from the experimental setting used by Ferrando and Anguiano-Carrasco (2011), that is made up of two groups surveyed in two different occasions (mixed repeated measure, between subject design); in this case we had a high level of researcher control but a low level of ecological validity. In the second example (study two), we used only two groups (control and experimental) with two different conditions: honest and faking-motivating. Finally, in the third example (study three), we tried to simulate a scenario somewhat opposite to the first study, i.e., a low level of researcher control and a high level of ecological validity. We used a neutral experimental condition that is we administered the research questionnaire to the respondents without any instruction, apart from the usual way to introduce a survey (e.g., privacy statements, anonimity issues, neither right nor wrong answer, etc.; Joint American Educational Research Association and others, 2014). In real life context, usually, we can only work on subjects' responses without knowing both their actual intentions as regards faking or not, and their actual degree of faking. Consequently, we need to hypothesize models of faking behavior and to compare them with empirical data (see for example Lombardi and Pastore, 2012, 2014).

### 3.1. Measure

In each of the following studies data consisted of participants' responses to 12 items of the Perceived Empathic Self-Efficacy Scale, Adult version (AEP/A; Caprara, 2001), scored on a 5-point scale where 1 denotes that she/he “Cannot do at all” the behavior described by the items, while 5 denotes that she/he “Certain can do” it. AEP/A is a unidimensional and easy-to-administer scale that was designed to assess individuals' perceived capability to recognize emotions, feelings, preferences, and needs of other people (e.g., “Understand the state of mind of others when you are very involved in a discussion?”). Anyway, we remind the reader that the main aim of this contribution was to test the validity of the SGR method, and we were not interested in analyzing or evaluating psychometric properties of the AEP/A scale.

The studies were projected and realized following all the ethical guidelines of the Italian Association of Psychology (AIP) and of the Ethic Committee of Psychological Research at Padua University. As participants in the studies were guaranteed complete anonymity and research presented no more than minimal risk to them, no personal identifiers were collected and only oral consent was obtained. Under these circumstances, and also giving the methodological nature of these studies, neither written consent nor the approval of Ethics Committees should reasonably be considered as something mandatory in itself. Respondents were requested to provide gender, age and, only in the case of the first study, to create a code formed by the first letters of name and surname and four digits representing month and day of birth (e.g., MP1026): this code was used only as a criterion to pair subjects across the two data collection occasions.

Moreover, everyone was told that participation was voluntary, without any form of compensation neither paid nor credited, and that all data would be treated for research purposes only. It was made clear to every participant that it was her or his right to “withdraw” or “opt-out” of the study or procedure at any time, and that return of questionnaires would be interpreted as expression of willingness to participate in the study. Afterwards, respondents were provided with all the necessary information on the nature, design and aim of the study. None retracted consent to participate.

### 3.2. R-SGR approach

In the first and second experiment matrix F represented data coming from the motivated-distorted condition. Data replacement process was applied to this matrix, following the three faking scenarios described earlier (i.e., uninformative, slight, and extreme). Subsequently, results were compared with data belonging to the D matrix, which represents the honest condition. On the contrary, in the case of experiment number three, we only had the F matrix, on which the replacement process was performed accordingly. The D type matrices were generated through a MC simulation process following *ad-hoc* hypotheses. Eventually, results were obtained by comparing observed-vs.-simulated data.

In particular, data analysis was focused on items' marginal means and, by means of Confirmatory Factor Analysis, on factor loadings and fit indices. Evidence on marginal means and fit indices could be linked to previous study (Lombardi and Pastore, 2012). All analyses were performed via the R software (R Core Team, 2015) using packages lavaan (Rosseel, 2012) and sgr (Lombardi and Pastore, 2014).

### 3.3. Study one

The aim of this study was to to apply the R-SGR procedure in the context of a experimental design such as that adopted by Ferrando and Anguiano-Carrasco (2011).

#### 3.3.1. Methods

##### 3.3.1.1. Participants, design, and procedure

A total of 444 undergraduate students from the School of Psychology at Padova University took part in the study on a voluntary basis, of which 93 were males and 351 females (mean age of 21.49, *s.d*. 3.95, ranging from 18 to 54). They were administered the AEP/A items in three classroom groups (ranging from 69 to 161 students) at two points in time during the lesson period, with a retest interval of about 6 weeks. At time 1 (*T*1) all participants were asked to respond honestly, since data would be used in order to validate the scale into the Italian context. At time 2 (*T*2), in each classroom students were randomly assigned to the two different conditions that defined the research design groups. The participants assigned to condition 1 (control group, CG) were retested using the same instructions used at *T*1. Participants assigned to condition 2 (experimental, faking-motivating condition group, FMG) were asked to imagine themselves as candidates applying for a job that they really wanted, and try to get the job. The job description led them to believe that the ability to deal with people and to work harmoniously in teams would be a decisive characteristic for the successful candidate.

There were 378 subjects at *T*1 and 314 subjects at *T*2, but only 248 students were present at both times; we also removed all subjects with missing data (12 subjects). The final sample was composed by 236 subjects, of which 46 were males and 190 females (mean age of 21.33, *s.d*. 4.06, ranging from 18 to 51). The control group (honest instructions at both times, CG) was made up of 66 and 170 belonged to the experimental group (honest instructions at *T*1 and faking-motivating instructions at *T*2, FMG). Although, the sample sizes were dissimilar, there were no significant differences as regards gender and age between the two groups.

##### 3.3.1.2. Preliminary analysis

First, we compared mean scores of the groups on the 12 items at *T*1 and found no difference (all comparisons were not statistically significant, Cohen's *d* ranging between 0.02 and 0.29). Then, we computed mean difference between *T*2 and *T*1 for each group. For CG differences between means ranged from −0.3 to 0.17 (all comparisons were not statistically significant, Cohen's *d* ranging between 0 and 0.33); for FMG differences between means ranged from 0.15 to 0.47 (all comparisons were statistically significant, Cohen's *d* ranging between 0.16 and 0.63). These results indicate that FMG subjects modified their answers in the hypothesized direction (toward higher values) and this supports the fact that the experimental manipulation worked as expected.

##### 3.3.1.3. Invariance analysis

**Figure 3:**
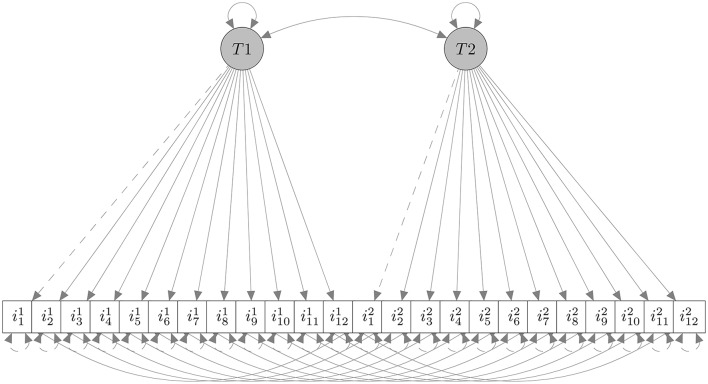
**The hypothesized factor model**. $i_{i}^{1}$, *i* = 1, 2, …, 12 are the 12 items at *T*1; $i_{i}^{2}$, are same items at *T*2. The invariance analysis was performed only at *T*1.

As a second step, we performed a unidimensional Confirmatory Factor Analysis using the 12 items collected at *T*1 (i.e., the factor *T*1 in Figure 3), and tested factorial invariance across the two groups of respondents at the same time (*T*1). We used the Diagonally Weighted Least Squares estimator as suggested by Forero et al. (2009). In Table 1 results of invariance analysis, following a scheme suggested by Beaujean et al. (2012) are reported. The first two rows (Models 1a and 1b) are model fit for each group separately (Meade et al., 2008) which turned out to be satisfactory. Other models tested were configural invariance (2), the factor model is the same across both groups: $M^{CG}=M^{FMG}$), thresholds invariance (3), item thresholds are the same across both groups: τ^*CG*^ = τ^*FMG*^), metric invariance (4), factor loadings are the same across both groups: (τ, Λ)^*CG*^ = (τ, Λ)^*FMG*^), and invariance of factor variance (5), factor variances are the same across both groups: (τ, Λ, Φ)^*CG*^ = (τ, Λ, Φ)^*FMG*^), respectively. Results supported invariance of the factor model across groups at *T*1.

**Table 1:** **Study one: Fit Statistics for Invariance Testing at ***T***1: 1a and 1b = models for each group separately; 2 = Configural invariance; 3 = Thresholds invariance; 4 = Metric invariance; 5 = Factor variance invariance**.

	***n***	**χ^2^**	**df**	***p***	**Δχ^2^**	**Δdf**	**Δ*p***	**CFI**	**NNFI**	**RMSEA**
1a. CG (honest)	66	39.76	54	0.926				1.000	1.215	0.000
1b. FMG (faking)	170	67.02	54	0.110				0.931	0.916	0.038
2. $M^{CG}=M^{FMG}$	236	105.99	108	0.537				1.000	1.010	0.000
3. τ^*CG*^ = τ^*FMG*^	236	133.91	135	0.510	27.93	27	0.415	1.000	1.004	0.000
4. (τ, Λ)^*CG*^ = (τ, Λ)^*FMG*^	236	135.26	146	0.728	1.34	11	1.000	1.000	1.038	0.000
5. (τ, Λ, Φ)^*CG*^ = (τ, Λ, Φ)^*FMG*^	236	135.08	147	0.750	0.17	1	0.677	1.000	1.041	0.000

##### 3.3.1.4. Factorial model

Figure 3 represents the factorial model considered in our analysis. The model contained 12 items loading on a single factor at *T*1 and the same 12 items (repeated measures) loading on the same single factor ad *T*2. Given the model's invariance at *T*1 (see Table 1), we expected a difference between CG (same instructions at *T*1 and *T*2) and MFG (faking-motivating instructions at *T*2) in the complete model. In CG we obtained the following fit indices: CFI = 1, NFI = 0.852, NNFI = 1.253, RMSEA = 0, indicating the satisfactory goodness-of-fit of the model. In FMG we obtained the following fit indices: CFI = 1, NFI = 0.926, NNFI = 1.019, RMSEA = 0, and even this model showed a good fit. The only relevant difference between fit indices was related to NFI values, that we already know to be the index most sensitive to fake perturbation (see Lombardi and Pastore, 2012, 2016). Consequently we applied the R-SGR procedure on FMG data, in order to obtain results similar to those observed in CG.

#### 3.3.2. R-SGR analysis

Subjects of FMG increased their scores in the faking-motivating condition (at *T*2), consequently we considered only responses of these subjects at *T*2 and adopted a replacement model in which responses were exclusively lowered with π as probability of replacement. We defined three faking scenarios: *uninformative* setting SGR parameters to γ = 1 and δ = 1; *slight*, γ = 4 and δ = 1.5; *extreme*, γ = 1.5 and δ = 4. The probability of faking was set to π ∈ {0.25, 0.5, 0.75, 1}.

In sum, we used the items responses of FMG (as F matrix) and on this data-matrix the following procedural steps were repeated 2000 times for each of the 3 (faking scenarios) × 4 (values of π) = 12 combinations:

1. Replaced a percentage of π × 100 items responses at *T*2 (items $i_{i}^{2}$, *i* = 1, 2, …, 12, in Figure 3) depending on fake scenario (*uninformative, slight*, and *extreme*).
2. Computed marginal means of items obtained by replacements.
3. Tested the factorial model, estimating model parameters, and fit indices.

The whole procedure generated a total of 2,000 × 3 × 4 = 24,000 new data matrices as well as an equivalent number of model parameters and fit indices. Replications that did not converge or produced improper solutions were excluded; a solution was deemed improper when at least one estimated variance was negative or factor loading was larger than 10 in its absolute value. In total we excluded 1,016 replications (4% of the total), 384 in the uninformative condition, 4 in the slight and 628 in the extreme, respectively; consequently, in each experimental conditions remained a number of replications varying from 1,726 to 2,000.

We used the following statistic, the Average Relative Bias (ARB), in order to estimate the bias for the 12 experimental conditions:

$$\begin{array}{l} ARB=100\left(1/B\right)\overset{B}{\underset{b=1}{\sum }}\left(1/V\right)\overset{V}{\underset{v=1}{\sum }}\left(\frac{{\hat{\theta }}_{bv}-\theta_{v}}{\theta_{v}}\right) \end{array}$$

with ${\hat{\theta }}_{bv}$ and θ_*v*_ being the *v*-element of the reconstructed statistic (marginal mean, factor loadings, or fit indices) in the *b*-sample replicate (*b* = 1, 2, …, *B*), and the *v*-element of the observed statistic (i.e., empirical marginal mean, factor loadings, or fit indices), respectively. In general, a large absolute value of the ARB measure (e.g., |45.07|) is an indication that a large discrepancy between the two statistics (observed and reconstructed) has occurred (e.g., 45.07%) and, conversely, the lowest the ARB value (e.g., |0.11|) the best the outcome in terms of the reconstructed statistic. Cut off values for the ARB depends on the study context. For example, if the ARB is used in Monte Carlo simulations, a relative bias values <5% may indicate a trivial bias, values between 5% and 10% may indicate a moderate bias, and values >10% may indicate a substantial bias (e.g., Kaplan, 1989; Curran et al., 1996; Yang-Wallentin et al., 2010). In the context of the present research, which is not only focused on Monte Carlo simulations, we will both follow these cutoff values in order to interpretate the overall results, but, adopting an exploratory perspective, we will also consider ARB values slightly above these limits.

#### 3.3.3. Results

##### 3.3.3.1. Marginal means

First we examined marginal means obtained in reconstructed data (FMG items at *T*2). In Table 2 (first three rows) are reported ARB values computed for each of the 3 × 4 = 12 experimental conditions. The left columns represent the ARB values computed with respect to CG at *T*2 (i.e., same occasion, independent groups); the right columns represent values computed respect to FMG at *T*1 (i.e., same subjects, different occasions). The 0 columns represent the ARB value based on difference between the observed marginal means (the percentage of data replaced is zero); in both cases the value was positive (6.74 and 5.8) thus supporting the hypothesis that faking-motivating condition induced FMG subjects to increase values in their responses at *T*2, therefore obtaining mean values higher than those observed at *T*1, and also higher than those of CG at *T*2. In all other cases the data reconstruction produced lower mean values (i.e., negative values of ARB) compared to those observed in the two conditions (CG at *T*2 and FMG at *T*1). In the slight scenario with π = 0.25 we observed the absolute lower ARB values (−0.11 and −0.99, respectively).

**Table 2:** **Study one: ARB for marginal means, factor loadings, and NFI as a function of percentage of replacements (π × 100) and fake scenarios, respect to CG (left side) and to FMG (right side)**.

	**CG**					**FMG**				
	**0**	**25**	**50**	**75**	**100**	**0**	**25**	**50**	**75**	**100**
**MARGINAL MEANS**										
Uninformative	6.74	−3.11	−12.94	−22.76	−32.61	5.80	−3.96	−13.70	−23.44	−33.19
Slight	6.74	−0.11	−6.94	−13.78	−20.63	5.80	−0.99	−7.76	−14.54	−21.33
Extreme	6.74	−6.09	−18.93	−31.77	−44.60	5.80	−6.92	−19.64	−32.36	−45.07
**FACTOR LOADINGS**										
Uninformative	−3.57	3.16	13.44	10.51	10.76	−18.15	−12.46	−3.88	−6.81	−6.66
Slight	−3.57	1.83	12.85	16.39	6.32	−18.15	−13.58	−4.32	−1.52	−10.04
Extreme	−3.57	1.96	−18.31	−22.89	8.73	−18.15	−13.36	−30.34	−35.43	−9.00
**NFI**										
Uninformative	8.76	−7.22	−19.92	−24.28	−23.81	0.00	−14.69	−26.37	−30.37	−29.94
Slight	8.76	−0.13	−7.75	−10.31	−4.51	0.00	−8.17	−15.18	−17.53	−12.20
Extreme	8.76	−13.00	−26.08	−27.72	−23.65	0.00	−20.00	−32.03	−33.53	−29.80

*Marginal means and factor loadings are referred to CG at T2 and to FMG at T1, respectively*.

**Figure 4:**
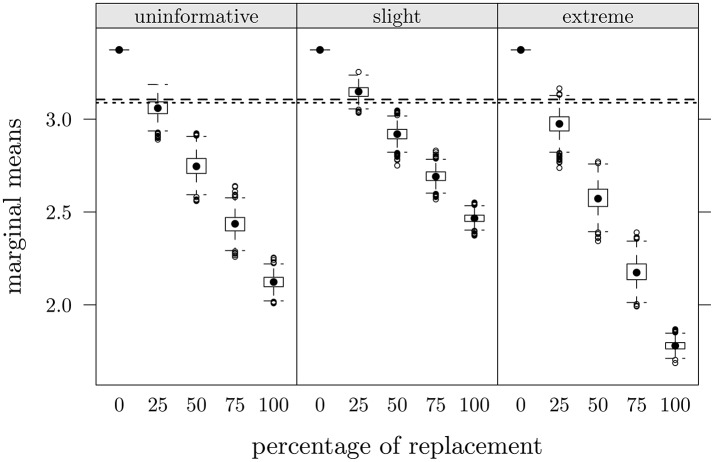
**Study one: Item 3 marginal means as a function of percentage of data replacements in the three scenarios**. Dashed lines represent observed mean values in CG at *T*2, dotted lines represent observed values in FMG at *T*1.

Since marginal means as a function of percentage of data replacements in the three faking scenarios were similar for all items, Figure 4 depicts only the case of item 3, as an example. Dashed lines represent observed means in CG at *T*2 and dotted lines observed means in FMG at *T*1. It seems evident that with an increase of the percentage of data replacements the marginal means of items decrease, consistently with expectations; moreover it is possible to note the low variability of distributions. In approximately all items, with 25% of data replacement (π = 0.25), we observed the lowest differences between original and reproduced means: in this condition, the average differences between the mean values observed and those produced by replacement were the lowest and were equal to 0.01 for CG (at *T*2) and 0.04 for FMG (at *T*1).

##### 3.3.3.2. Factor loadings

Subsequently, we examined factor loadings of the *T*2 factor (i.e., relative to items $i_{i}^{2};i=1,2,\ldots ,12$ in Figure 3), obtained with reconstructed data. In Table 2 (rows from 4 to 6) are reported ARB values computed for each experimental condition. On the left side of the table ARB was computed with respect to observed loadings in CG at *T*2 (same items), and on the right side with respect to FMG at *T*1 (items $i_{i}^{1};i=1,2,\ldots ,12$). Negative values in π = 0 condition means that observed loadings in FMG at *T*2 were lower than corresponding loadings in CG at the same time and in FMG at *T*1. In the CG the minimum value corresponded to a slight scenario with 25% of replacements (ARB = 1.83), then we observed other low values in the others scenarios at same percentage of replacements (ARB = 3.16 and 1.96, respectively) and similar values in the slight and extreme with 100% of replacements (6.32 and 8.73, respectively). In the FMG the lowest values were those of the slight scenario with 75% of replacements (ARB = −1.52), and those of the uninformative and again slight scenario with 50% of replacements (−3.88 and −4.32, respectively). Nonetheless, the patterns of ARB values were quite similar in both groups: in the uninformative and the slight scenario values increased up to 50 and 75% of replacements, respectively, in the extreme scenario values alternatively decreased and increased.

**Figure 5:**
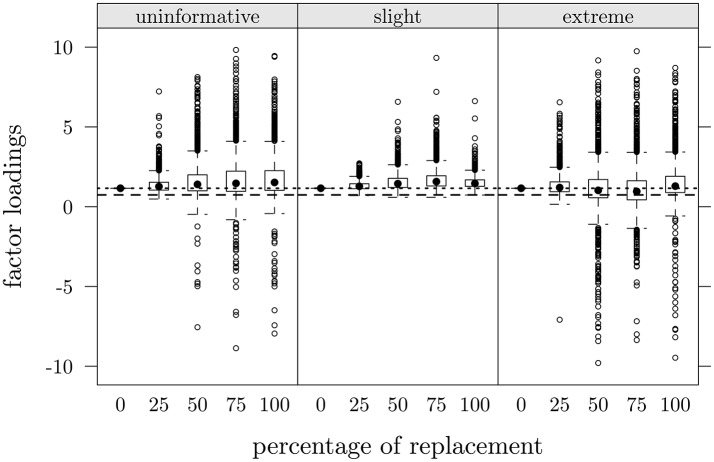
**Study one: Item 3, factor loadings at ***T***2 as a function of percentage of data replacements in the three scenarios**. Dashed lines represent observed values in CG at *T*2, dotted lines represent observed values in FMG at *T*1.

Similarly to Figure 4, Figure 5 depicts loadings distribution as a function of percentage of data replacements (π × 100) only for item 3 at *T*2 in the three scenarios. Dashed lines represent the loading's value observed in CG (at *T*2), dotted lines represent the loading's value in FMG for item 3 at *T*1.

Loadings in reconstructed data ranged from 0.01 to 9.77, which a variability increase as a function of percentage of replacements. Interestingly enough, despite this variability increase, a relationship between percentage of replacements and expected values of parameter estimates did non emerge clearly: by regressing loadings values on percentage of replacements we obtained *R*^2^ ranging from 0.0001 to 0.03.

##### 3.3.3.3. Fit indices

**Figure 6:**
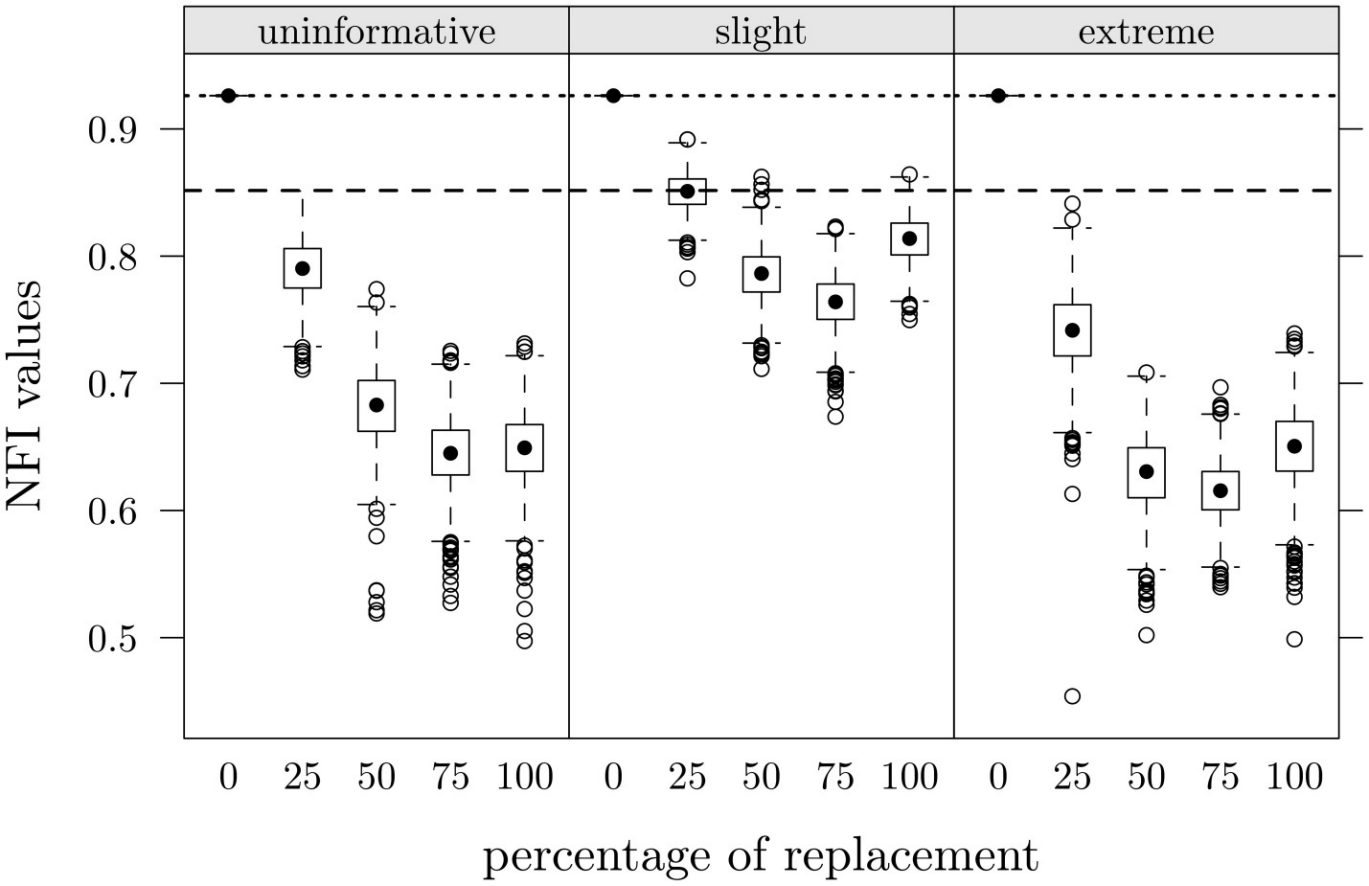
**Study one: NFI values as a function of percentage of data replacement in the three different scenarios**. Dashed lines represent observed value in CG, dotted lines represent observed value in FMG.

Finally, we considered the Normed Fit Index of the factorial model. NFI resulted as the fit index which may be more influenced by fake data (see Lombardi and Pastore, 2012, 2016), and even in this case it was the index that presented the greatest differences between the two groups (i.e., 0.852 for CG and 0.926 for FMG). In Table 2 (last three rows) we report the ARB values of this index calculated for each experimental condition. On the left side of the table the ARB was computed with respect to the observed NFI in CG, on the right side with respect to the observed NFI in FMG. Positive values in π = 0 condition for CG mean that the observed NFI in this group was higher than the value observed for FMG. For both groups the slight scenario with 25% of replacements showed the lowest ARB value (−0.13 and −8.17, respectively); all patterns were similar for the three faking scenarios: ARB values decreased up to the 75% of data replacement. In Figure 6 are depicted NFI distributions as a function of percentage of replacement in the three scenarios; dashed lines represent the NFI value observed in CG, dotted lines the observed value in FMG. The slight scenario produced NFI values which were closer to the observed values in CG. The general trend is very similar to that reported by Lombardi and Pastore (2012, 2016).

##### 3.3.3.4. Conclusions

The aim of this study was both to replicate the experimental design by Ferrando and Anguiano-Carrasco (2011) and also to apply, without any contribution coming from social desirability measures, the R-SGR procedure. The experimental manipulation was successful: the average response of participants in the FMG condition at T2 was both higher than that of the same respondents at T1, and it also turned out to be higher than that of participants in the CG condition. With no information as regards the actual motivation or the tendency of participants to adjust their answers, three fake scenarios (i.e., uninformative, slight, and extreme) and four percentages levels of scores replacement (i.e., 25, 50, 75, 100) were hypothesized and implemented.

The study showed that the higher the percentage of faking, the lower the marginal means; also, the higher the faking the higher the observed variability of factor loadings even if without any substantial consequence as concerns expected values and, as regards the NFI index, its values decreased up to a constant percentage of fake within all the scenarios. Finally, of all the faking scenarios here hypothesized, it was the slight one, along with 25% of replacement, which determined the lowest possible distance between data matrices coming from the FMG and the CG, respectively. Generally speaking, this means that it would be possible to reconstruct an estimated D matrix—which was supposed to be made of not-distorted answers—by applying a slight negative shift to about 25% of the F matrix, which contained fake-distorted answers. In practice, assuming that subjects in the FMG condition adopted a slight alteration strategy of their responses, 25% represents the most likely estimation of fake among the percentage here implemented. In fact, in this scenario, ARB values for both marginal means and NFI are those closest to zero.

### 3.4. Study two

The second study aimed to replicate the results of the first study using data collected only once.

#### 3.4.1. Methods

##### 3.4.1.1. Participants, design, and procedure

A total of 494 undergraduate students from the School of Psychology at Padova University took part in the study on a voluntary basis, of which 103 were males and 391 females (mean age of 20.64, *s.d*. 2.72, ranging from 18 to 48). They were administered the same AEP/A scale used in study one (in 7 classroom groups ranging from 7 to 145 attendees) during the lesson period. The scale was administered anonymously in a paper and pencil format. The respondent had to provide only gender and age. All participants were asked to follow instruction reported in the front page of the questionnaires. Unbeknownst to the subjects, two different kinds of instructions were randomly assigned: (1) control/honest and (2) faking-motivating, which were exactly the same used in study one. Participants assigned to condition 1 (Control Group, CG) were 243; participants assigned to condition 2 (Faking-Motivating Group, FMG) were 251. No significant differences as regards gender and age between the two groups emerged.

##### 3.4.1.2. Preliminary analysis

We compared the observed means of the 12 items for each group. As expected, subjects of FMG obtained higher means for all items with differences ranging from 0.1 and 0.3. Based on this preliminary observation, we hypothesized, even in this second study, that subjects in the FMG condition modified their answers. In this case we had only a single administration, consequently the factorial model is a single-factor model with 12 indicators correspondig to the 12 items of the AEP/A scale, that is the same as factor *T*2 in Figure 3. In FMG we obtained the following fit indices: CFI = 0.954, NNFI = 0.944, RMSEA = 0.064, NFI = 0.914, supporting goodness of fit of the model. In CG we got the following fit indices: CFI = 0.928, NNFI = 0.912, RMSEA = 0.066, NFI = 0.87, substaining adequacy of the model. Again, NFI was higher for FMG compared to CG.

#### 3.4.2. R-SGR analysis

We observed that the faking-motivating instructions led subjects to increase the scores of their answers. As in study one, our aim was to estimate the impact of faking, starting from FMG data and adopting the same three scenarios: *uninformative* setting SGR parameters to γ = 1 and δ = 1, *slight* γ = 4 and δ = 1.5, *extreme* γ = 1.5 and δ = 4. The probability of faking was set to π ∈ {0.25, 0.5, 0.75, 1}, with the constrain of allowing only replacements with lower values than those observed. We then used the data matrix of FMG (which was our F) and repeated for 2,000 times for each of the 3 (scenarios of faking) × 4 (values of π) = 12 combinations the following steps:

- Replaced a percentage of π × 100 responsens on the 12 items in the F matrix depending on fake scenario (*uninformative, slight*, and *extreme*).
- Computed marginal means of items obtained by replacement.
- Tested the factorial model, estimating parameters and fit indices.

The whole procedure generated a total of 2,000 × 3 × 4 = 24,000 new data matrices as well as an equivalent number of model parameters and fit indices. Replications that did not converge or produced improper solutions were excluded: i.e., 856 in the uninformative condition, 21 in the slight and 1,425 in the extreme, respectively (in total about 10% of replications were deleted). Consequently, in each experimental conditions a number of replications varying from 1,532 to 2,000 remained. Adopting the same strategy of study one, ARB was used in order to estimate the bias for the 12 experimental conditions comparing the reconstructed-distribution based results with those observed in the CG.

#### 3.4.3. Results

##### 3.4.3.1. Marginal means

The patterns of means for all items were similar to those observed in study one (see Figure 4). In Table 3 (first three rows) are reported ARB values for each of the 12 experimental conditions. The value for π = 0 (5.69) indicates that marginal means observed in FMG are higher than those in CG. In the slight scenario we observed lower values compared all the others scenarios; in particular the condition with 25% of data replacements showed the absolute lowest value (−3.68). The decrease of ARB values as a function of percentage of fake indicates that marginal means of items decrease.

**Table 3:** **Study two: ARB for marginal means, factor loadings, and NFI as a function of percentage of replacements (π × 100) and fake scenarios**.

	**0**	**25**	**50**	**75**	**100**
**MARGINAL MEANS**					
Uninformative	5.69	−7.50	−20.70	−33.92	−47.10
Slight	5.69	−3.68	−13.04	−22.43	−31.79
Extreme	5.69	−11.32	−28.37	−45.42	−62.41
**FACTOR LOADINGS**					
Uninformative	−25.03	−8.89	−19.90	−48.90	−35.86
Slight	−25.03	−14.53	−10.80	−9.88	−17.04
extreme	−25.03	−7.81	−58.14	−89.84	−84.80
**NFI**					
Uninformative	4.99	−14.06	−38.79	−51.57	−47.42
Slight	4.99	−3.94	−12.56	−14.91	−6.72
Extreme	4.99	−24.01	−54.13	−60.50	−59.32

##### 3.4.3.2. Factor loadings

In Table 3 (rows from 4 to 6) are reported ARB computed for factor loadings. As in study one, factor loadings presented many differences in ARB values among the experimental conditions; negative values in the condition with 0% of replacements means that observed loadings in FMG were lower than the corresponding loadings in CG. The lowest absolute ARB values were obtained in the uninformative and in the extreme scenarios with 25% of data replacements (−8.89 and −7.81, respectively), followed by the values of the slight scenario with 75% and 50% (−9.88 and −10.8). However, in the last one, we observed, on average, the lowest possible ARB values. As in the first study, the expected value of the loadings seemed not to be particularly influenced by the percentage of faking: by regressing loadings values on percentage of replacements we obtained *R*^2^ values ranging from 0.01 to 0.06.

##### 3.4.3.3. Fit indices

The last three rows of Table 3 report ARB values of NFI for each experimental condition. Positive value in π = 0 condition reflects the fact that observed NFI was higher in FMG than in CG. The general trend in all scenarios is similar to that observed in study one (see Table 2, last three rows), with a decrease of ARB values up to 75% of data replacements. The slight scenario presented the lowest ARB values, with the absolute minimum in the π = 0.25 condition (ARB = −3.94).

##### 3.4.3.4. Conclusions

The aim of the second study was to replicate the results of the first study with a lower degree of experimental control, thus going in the direction of a more ecological situation. In particular, no repetition of measures took place. Again, the experimental manipulation was successful: the average response of participants in the FMG condition was higher than that of participants in the CG condition. Similarly to study one, three fake scenarios were implemented (i.e., uninformative, slight, and extreme) along with 4 percentages levels of scores replacement (i.e., 25, 50, 75, 100).

R-SGR allowed to replicate the results of study one as regards marginal means, factor loadings and the NFI index. This fact emerges clearly if Table 3 and the left side of Table 2 are compared. In practice, among the three replacement scenarios here considered, the slight one presented the narrowest ARB ranges. Moreover, within this scenario, percentages closest to zero are observed at 25%. Consistently to study one, this percentage represented the most likely estimation of fake among those implemented.

### 3.5. Study three

The aim of the study was to reproduce results of both study one and two. In this case, data were collected only at one point in time and no specific instruction was assigned to participants.

#### 3.5.1. Methods

##### 3.5.1.1. Participants, design, and procedure

A total of 126 undergraduate students from the School of Psychology at Padova University volunteered for the study. Males were 29 and females were 97 (mean age of 19.71, *s.d*. 0.94, ranging from 19 to 23). They answered the AEP/A scale in a single classroom during the lesson period. The scale was administered anonymously in a paper and pencil format and the respondent had to provide only gender and age. All participants were asked to fill out the questionnaire without any kind of additional instructions, a part from the standard introduction for survey's administration (e.g., Joint American Educational Research Association and others, 2014). By doing this we tried to reproduce a real-life context in which it could only be hypothesized that some subjects would probably distort their responses while others would not.

#### 3.5.2. R-SGR analysis

Unlike study one and study two, in the current one we had only one observed data sample. Consequently, we generate the D matrix using an *ad-hoc* MC procedure in order to compare this data to those obtained from the replacement of the observed ones.

##### 3.5.2.1. Generative model

First, we defined a generative model for the unknown data matrix D based on the following prior hypotheses: (1) the response item distribution has a binomial form with parameter π = 0.5; (2) item correlations are medium, around ρ = 0.25. Our parameters were chosen after data inspection, in accordance with the hypothesis that response values should be higher for those subjects supposed to be more prone to faking vs. the supposed to be more honest ones. Actually, both marginal means (based on the binomial) and correlations should be lower than those observed (Lombardi et al., 2015); for the overall sample (*n* = 126) the grand mean of the observed item marginal means was 3.59 and mean of observed correlations was 0.24 (with *s.d*. 0.11). In sum, this generative model represents our hypothesis on D: namely, that items responses were symmetrically distributed around 3, and that they were also correlated to each other only at an average level. The hypothesis on D could be easily adapted based on researcher's hypotheses or prior knowledge (e.g., normative parameters of cognitive, aptitude, or personality tests; results of comparable previous study, when available).

##### 3.5.2.2. Data generation and replacement

For each of the combinations of the three scenarios of faking and the five values of π, we repeated the following algorithmic steps for 2,000 times:

- Generated a raw-data set D with size 126 × 12 using the single factorial model and parameters θ_*D*_ = (π = 0.5, ρ = 0.25).
- Computed marginal means of items, tested the factorial model (including estimated parameters) and fit indices.
- Replaced a percentage of π × 100 responses on the 12 items in the F matrix depending on the fake scenario (uninformative, slight, and extreme).
- Computed marginal means of items obtained by replacements, tested the factorial model (including estimated parameters) and fit indices.

The whole procedure generated a total of 2,000 × 3 × 5 = 30,000 new data matrices D, and 30,000 reconstructed data matrices as well as an equivalent number of model parameters and fit indices. Replications that did not converged or produced improper solutions were excluded: in total 73 replications of the data generation process (i.e., 0.24%) and 2,073 of the data replacement process (i.e., 6.91%), of which 726 in the uninformative condition, 20 in the slight and 1,327 in the extreme, respectively. Consequently, in each experimental condition a number of replications varying from 1,513 to 2,000 was used. Even in this study we made reference to the ARB in order to estimate bias. Unlike the previous studies, in which we had a control group, in the current one the produced (reconstructed-distribution based) results were compared to the corresponding estimated values obtained by generating data with parameters θ_*D*_. In particular we considered as control values marginal means of items, factor loadings, and fit indices obtained from the data generation process.

#### 3.5.3. Results

##### 3.5.3.1. Marginal means

In Table 4 (first three rows) are reported ARB values for each of the 12 experimental conditions. Positive value (19.74) in the 0 condition indicates that observed marginal means are higher than those generated in the D matrices. The lowest ARB values were those of the extreme scenario with 25% of replacements (0.58), of the slight with 50% (−1.68) and of the uninformative with 25% (4.82). However, the slight scenario showed, on average, values closer to those referred to empirical data. Even in this case, we observed, for all scenarios, that items marginal means decreased as a function of percentage of replacements.

**Table 4:** **Study three: ARB for marginal means, factor loadings, and NFI as a function of percentage of replacements (π^**−**^ × 100) and fake scenario**.

	**0**	**25**	**50**	**75**	**100**
**MARGINAL MEANS**					
Uninformative	19.74	4.82	−10.15	−25.08	−40.08
Slight	19.74	9.03	−1.68	−12.40	−23.11
Extreme	19.74	0.58	−18.69	−37.81	−57.01
**FACTOR LOADINGS**					
Uninformative	−2.67	9.54	1.90	−25.54	−16.34
Slight	−2.67	−0.40	1.85	0.85	−4.71
Extreme	−2.67	11.84	−47.89	−84.27	−81.59
**NFI**					
Uninformative	7.12	−14.47	−37.06	−46.22	−42.59
Slight	7.12	−2.78	−12.76	−14.66	−4.18
Extreme	7.12	−25.11	−50.18	−55.53	−54.11

##### 3.5.3.2. Factor loadings

In the rows from 4 to 6 of Table 4 are reported ARB computed for factor loadings. Negative value in condition π = 0 (−2.67) indicates that simulated loadings are lower than the empirically estimated ones in our sample. The slight scenario presented the lowest ARB values in general (the absolute lower value in the condition with 25% of data replacements was −0.4), while the slight and the extreme scenarios showed higher values. Also in this case there was a considerable variability in the distributions of factor loadings which, however, did not seem to affect the expected value of the estimates.

##### 3.5.3.3. Fit indices

In the last three rows of Table 4 are reported ARB values based on NFI for each experimental condition. Once again, the slight scenario resulted closest to the control condition showing the lowest value in the 25% data replacements condition (−2.78). Even in this case, NFI's values decreased up to 75% of replacements thus supporting results of the other two studies.

##### 3.5.3.4. Conclusions

The aim of the third study was to reproduce results of both study one and two, even in the case where the researchers were not able to identify subjects' motivation or propension toward faking.

Obviously this condition, which could be seen as much more realistic of the previous ones, requires a special attention in terms of hypotheses related to the D data matrix, since the results would be strongly dependent from the selected parameter values, in a way that recalls the concept of Bayesian priors. Additionally, this condition may also determine results which are both more complex and difficult to understand and interpret.

On the basis of the assumptions made on the generative model of D, which were both cautious and general, also in this case the application of R-SGR leads us to conclude that, on average, the closest scenario to the observed data was the slight one, which showed the narrowest ARB ranges. Overall, as regards percentage of replacement we concluded that the more appropriate one fell between 25% and 50%. However, in order to derive more practical conclusions, this result, which depends from the choosed generative model, should be compared to some other scenarios, defined on the basis of other generative models and linked to specific hypotheses.

## 4. Discussion and conclusions

In this paper we presented three applications of the SGR approach to fake data analysis issues thanks to the R-SGR mechanism. Since we used both samples coming from the same population (i.e., Psychology students at Padua University) and the same instrument (i.e., the AEP/A scale), we maintain that it is possible to compare results and to draw some useful conclusions and remarks supporting new research developments. By using ARBs, we tried to identify under which condition data obtained from reconstruction resulted closer to empirical data. It should be remembered that the lower the absolute ARB value, the more the two conditions under examination could be considered as similar to each other. In practice, when the range of ARB values is both narrowed and as close as possible to zero, we could assume to have identified a plausible scenario of faking.

**Figure 7:**
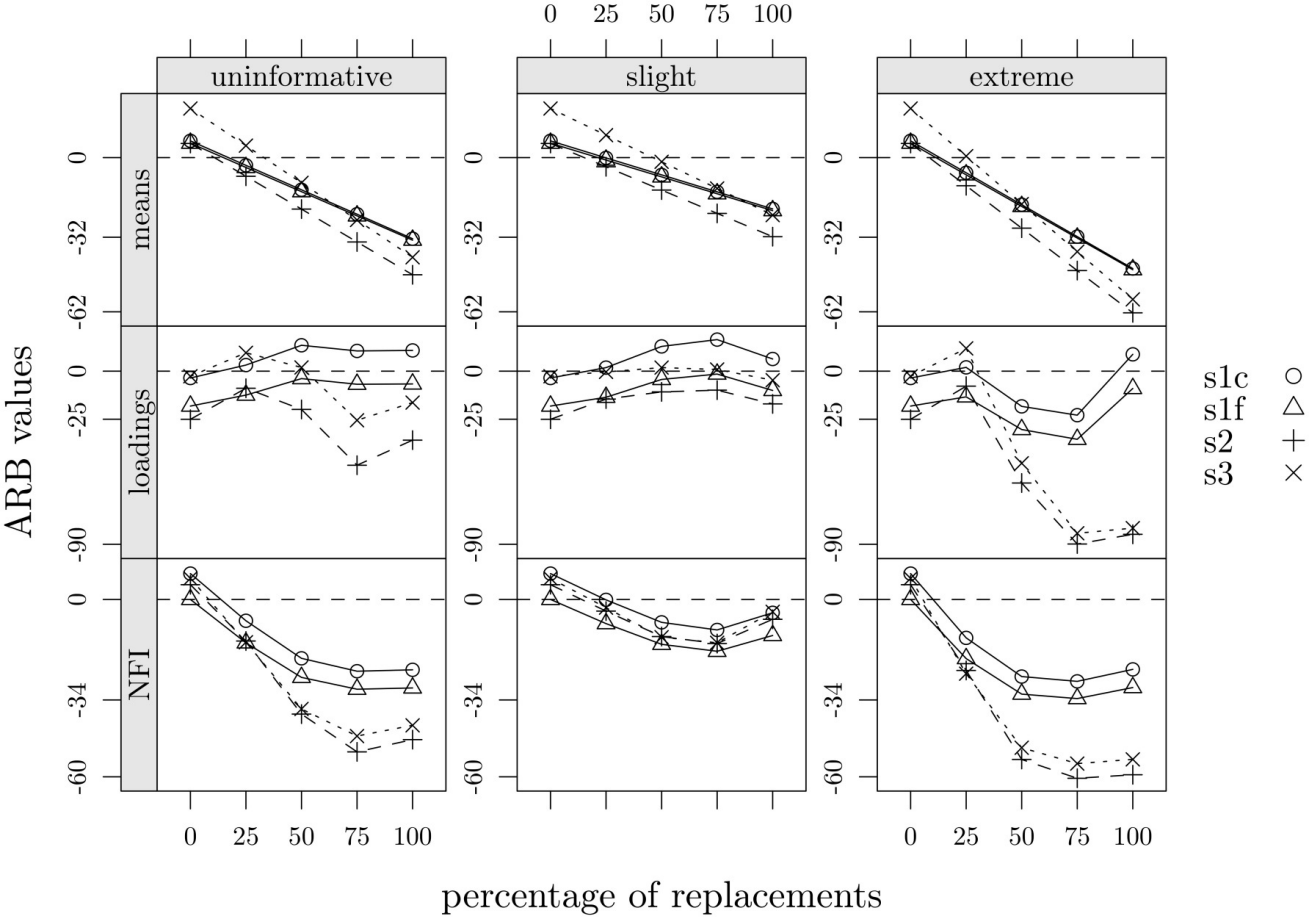
**Summary of ARB values as a function of percentage of replacements, faking scenarios, and statistics: s1c, study one, control group; s1f, study one, fake-motivating group; s2, study two; s3, study three**.

Overall, we observed that: (1) marginal means of items decreased as a function of percentage of data replacement, a consequence that largely rely on the substitution strategy here adopted. Furthermore, the slight scenario was, on average, the one which returned the lowest ARB values in all cases (−7.35 in study one, −13.05 in study two and −1.69 in study three; see Tables 2–4 and Figure 7); (2) factor loadings showed a variability increase which also went along with the percentage of data replacement; additionally, it was mainly the slight scenario that showed the ARB values closer to those observed; (3) as regards the values of the NFI index, they decreased as a function of the percentage of data replacement but only up to about 75% of data replacements, also reproducing the same pattern across the three scenarios. In sum, we concluded that, for all three studies, the scenario which best matched the observed data was the slight one, along with a percentage of replacement of about 25%. That is, the supposed-to-be fake-uncorrupted data matrix could be acceptably reconstructed by implementing a slight negative shift to about 25% of answers of the supposed-to-be fake-corrupted data matrix. Moreover, in the slight scenario there were no noticeable differences among the three experimental designs. As concerns the other two scenarios (uninformative and extreme) differences emerged between study one and both study two and three, particularly in the case of NFI and factor loadings, and especially when percentage of replacement approached or went beyond 50%.

On the basis of both the assumptions and the scenarios of faking here operationalized, we believe that important evidence on how the SGR approach could be used in order to study the behavior of some descriptive statistics and parameters under fake conditions was achieved, even in the case where neither a control group nor an appropriate control scale was available to the researcher or the practitioner. In fact, in real life contexts, such as when a recruitment and selection process is organized by a public or private company, just one group of individuals is answering to a test battery or to a questionnaire, and it could reasonably be assumed that only some of the candidates would somehow distort their answers in order to get the job. In this case, by setting specific hypotheses through which the generative model could be implemented (see study three), data analysis could be run by means of the R-SGR but not, at least to our knowledge, via other approaches or methodologies discussed in this paper.

Hypotheses or assumptions for data modeling could be derived from previous administration of the test/questionnaire or from previous studies (in our case, we could have based the hypotheses for the third study on the information coming from study one and two), following the same rationale behind the Bayesian prior distributions. Or they could also be derived from the norms of the instrument (e.g., when a renowned psychological test is administered), or from explicit expectations in line with personality theories or motivation theories (i.e., appropriately formalized by identifying and setting both γ and δ values). Moreover, the percentage of data replacement could be limited to a certain interval, such as lower or equal to 50%: actually, above that limit, it could be contended that the whole amount of fake data would be seriously threatening the validity of the entire dataset. Finally, in line with the results coming from the present paper, effects of the percentage of data replacement should be more carefully analyzed only within the interval ranging from 1% to 30%.

Among the limitations of the SGR approach we should certainly highlight its computational aspects: indeed, the time needed for data processing greatly increases as a function of both the number of replicas, the scenarios and the complexity of the statistical model to be tested. Another limitation is that SGR could give rise to difficulties of interpretation, since the behavior of some statistics or parameters under perturbation/reconstruction process might be difficult to grasp (see the case of factor loadings). It is easy to see that the SGR representation is different from other statistical perspectives which are more focused to solve the fake identification problem using specific *ad-hoc* empirical paradigms such as, for example, coached faking or ad-lib faking (e.g., Ferrando and Anguiano-Carrasco, 2011). In addition, SGR also differs from RR (*Randomized Response*; Chaudhuri and Mukerjee, 1988), which aims to estimate true responses thanks to randomization in order to encourage honest reports. Finally, we argue that SGR may fruitfully integrate information or results obtained using other statistical techniques like, for example, RR and that new important SGR models may stem from the necessity to deal with different problems beyond those considered in this study (i.e., the case of different typologies of data or measurement scales and/or different probabilities of faking across individuals or items).

The results of the three interconnected studies constituting the present paper must be considered as essentially descriptive and exploratory: both scenarios of faking and replacement percentages were mainly selected with the intent to investigate both potentiality and flexibility of the SGR approach. Quite honestly, very naïve assumptions as regards the answers to a psychological instrument were considered (e.g., data replacement was performed separately on each item belonging to the AEP/A scale, that is independently from all the other items), and this is certainly a limitation of the conclusions that could be drawn from our studies. However, SGR showed that it could be applied regardless of any statistical model (see, Pastore and Lombardi, 2014; Lombardi et al., 2015) and research design, and therefore it could be seen as a useful tool compared to other methods for all real life or realistic empirical situations.

## Ethics statement

This study was carried out in accordance with recommendations of the Ethical Committee for Psychological Research at Padova University, with oral informed consent obtained from all subjects.

## Author contributions

All authors contributed equally to the study design and were involved in data collection, statistical analysis, writing of the manuscript, and approved the final version of the manuscript.

### Conflict of interest statement

The authors declare that the research was conducted in the absence of any commercial or financial relationships that could be construed as a potential conflict of interest.

## Footnotes

^1^All technical information about SGR (e.g., the algorithm codes, the reference manual, and also several examples) is freely available online at https://cran.r-project.org/web/packages/sgr/index.html.
